# Healthcare professionals’ perspectives on the current state of obesity management training: A mix methods study

**DOI:** 10.1016/j.obpill.2025.100202

**Published:** 2025-08-19

**Authors:** Francisca Contreras, Tak Ying Louise Ko, Carel W. le Roux, Werd Al-Najim

**Affiliations:** Conway Institute, School of Medicine, University College Dublin, Belfield, Dublin 4, Ireland

## Abstract

**Introduction:**

Obesity is a disease that represents a major global health problem, affecting over a quarter of European adults and straining healthcare systems. Despite its multifactorial causes, many healthcare professionals lack adequate training and confidence in delivering effective, person-centered obesity care. Weight stigma and misconceptions further impair outcomes. Thus, improved education and interprofessional training are essential to support healthcare professionals in delivering optimal obesity care. The aim of this study is to explore the current state of obesity management training among healthcare professionals working in obesity management services by identifying perceived training gaps, confidence levels and impact on clinical practice.

**Methodology:**

This study employed a mixed-methods design by combining a cross-sectional survey of 100 healthcare professionals with semi-structured interviews of 20 professionals who work in obesity management. Survey data were analyzed descriptively, and interview transcripts underwent thematic analysis using Braun and Clarke's framework.

**Results:**

Of the 100 healthcare professionals surveyed, 88 % reported actively working in obesity management and completed the full survey. Undergraduate training in obesity care was limited, with only 44 % receiving any, and only 12 % rated it as good or excellent. Postgraduate training was pursued by 51 %, with only 6 % completing formal advanced education. Participant perceptions of obesity training were organized in 5 domains: a) Current state of obesity management training, b) Confidence level, c) Gaps in training, d) Impact on clinical practice and d) Recommendation for obesity training curriculum.

**Conclusion:**

Healthcare professionals report significant gaps in obesity management training that impact their confidence and clinical practice. The key recommendations made by participants in this study reflect both the perceived deficits and a clear demand for more structured and comprehensive training in obesity management. Integrating structured, evidence-based obesity education into healthcare training programs is essential to reduce stigma, build competence, and improve obesity care.

## 1 Introduction

Obesity is a disease that has developed as a critical global health challenge, with prevalence rates rising significantly across both developed and developing nations [1], except in the United States, where, according to the Centre for Disease Control and prevention (CDC), adult obesity rates declined slightly from 2017 to 2020 to 2021–2023 [2]. In Europe, over a quarter of adults are living with obesity, contributing to a growing burden on health systems and a rise in associated non-communicable diseases such as type 2 diabetes, cardiovascular disease, and certain cancers [3]. While the multifactorial nature of obesity, including biological, psychological, environmental, and socio-economic determinants, is increasingly acknowledged [4], a persistent mismatch remains between this complexity and the training healthcare professionals (HCPs) receive to manage the disease effectively [[5], [6], [7], [8]].

Multiple studies have highlighted widespread gaps in HCP education, with professionals reporting limited preparation in core areas such as behavioral counselling, pharmacotherapy, nutrition, and communication strategies tailored to patients living with obesity [6,[9], [10], [11]]. Confidence in delivering obesity care remains low, particularly among physicians and nurses who often lack structured training in long-term weight management, psychological support, or the coordination of multidisciplinary care [[12], [13], [14]]. Compounding these challenges is the continued presence of weight stigma and bias within clinical settings, which is frequently linked to poor patient experiences, reduced care engagement, and negative health outcomes [[15], [16], [17], [18]].

International research underscores the need for robust, competency-based obesity education frameworks. In the United States, the Obesity Medicine Education Collaborative (OMEC) has developed a set of comprehensive core competencies intended to guide undergraduate and postgraduate medical education [19]. Similarly, Obesity Canada has introduced a national framework of obesity education competencies focused on addressing weight bias, promoting a biopsychosocial model, and integrating the lived experience of patients [20]. These efforts are supported by the World Health Organization's Acceleration Plan to Stop Obesity, which calls for health professional training that equips clinicians to address social inequities and environmental determinants of obesity through a chronic disease lens [21].

However, there is a lack of research specifically focused on the training and confidence of professionals working in obesity management in Ireland. Understanding current state of obesity management training, perceived gaps, and the impact on clinical practice is essential for designing effective training interventions that support both HCPs and their patients.

### 1.1 Aim

The aim of this mixed-methods study is to explore the current state of obesity management training among healthcare professionals by identifying perceived training gaps, evaluating confidence levels and the impact of training on clinical practice among those working in the field of obesity care in a European country such as Ireland.

## 2 Methodology

### 2.1 Study design

This research employed a convergent mixed-methods design, integrating both quantitative and qualitative methodologies to comprehensively investigate training gaps in obesity management among healthcare professionals. Mixed-methods research is particularly suited to complex health education topics, as it provides a more complete understanding of data, allowing for both breadth and depth of insight [[22], [23], [24]].

The quantitative component used a descriptive survey design to gather numerical data regarding HCPs' self-reported training, perceived confidence, and clinical practices in obesity management. This approach allowed for the identification of patterns and generalizations across a broader population. Meanwhile, the qualitative component employed a descriptive and exploratory ethnographic design using semi-structured individual interviews. This approach facilitated in-depth exploration of participants' experiences, attitudes, and perceived challenges, aligning with an interpretivist epistemology, which seeks to understand meaning from the participants’ perspectives [22,23,25]. Semi-structured interviews were chosen for their flexibility and ability to obtain rich, narrative data while maintaining a consistent structure across interviews. These are especially useful in healthcare education research where complex, subjective experiences must be articulated and contextualized [25].

### 2.2 Participants and recruitment

#### 2.2.1 Survey

A cross-sectional survey was developed based on a comprehensive literature review. While no existing tool specifically addressed training for weight management professionals, elements from previously validated instruments were adapted to align with the study's objectives (see Appendix 1) [9,10,12,26]. The survey aimed to assess 100 respondents in areas such as education, confidence in obesity care, and perceived knowledge gaps.

#### 2.2.2 Interviews

A purposive convenience sample of 20 healthcare professionals working in obesity management in Ireland was recruited. These individuals had previously completed the online survey and indicated willingness to participate in follow-up interviews by answering the last question of the survey: “Would you be open to being contacted for a follow-up interview with the researcher to explore your knowledge and experience in greater detail?”. Those who responded positively were invited to provide their email address and were contacted by FC to conduct the interview.

### 2.3 Data collection

#### 2.3.1 Survey

The online survey was distributed using SurveyMonkey to healthcare professionals across Ireland involved in obesity management. Recruitment was supported by the professional networks of the research team and in collaboration with the Association for the Study of Obesity on the Island of Ireland (ASOI). Data collection occurred in November 2024.

#### 2.3.2 Interviews

Interviews were conducted over a three-month period, between November 2024 and January 2025. Following informed consent, obtained electronically via NitroSign, participants received a private Zoom link to attend one-on-one interviews with FC. An interview guide (Appendix 2) was used to ensure consistency across sessions. Interviews were audio-recorded and transcribed using Zoom's transcription feature under a University College Dublin (UCD) institutional license, in accordance with UCD and General Data Protection Regulation (GDPR) requirements.

The length of the interviews was ranged from 20 to 60 min, depending on participant engagement and depth of experience. For instance, professionals with extensive postgraduate training in obesity provided more detailed narratives, often resulting in longer interviews.

### 2.4 Data analysis

#### 2.4.1 Quantitative data

Survey responses were analyzed descriptively using Microsoft Excel. Frequencies and percentages were calculated and results presented in tables and figures. Descriptive interpretation focused on identifying key trends and patterns across the sample.

#### 2.4.2 Qualitative data

All interview transcripts were reviewed, cleaned, and anonymized by FC to remove identifiable information. The data were then imported into Maximum Qualitative Data Analysis (MAXQDA) 24, a professional software tool for qualitative and mixed methods research, where they were analyzed independently by two researchers (FC and LK) using reflexive thematic analysis following Braun and Clarke's six-phase framework [27]. This process involved: a) familiarization with the transcripts b) generating initial codes, c) searching for themes, d) reviewing themes, e) defining and naming themes, along relevant quotes, f) producing the report. The final stage involved collaborative discussion among the four authors (FC, LK, WA, ClR), who discussed the results of the previous five steps of the analysis, the thematic structure was finalized and interpretative insights were integrated to produce the final report (see Fig. 4).

This analytical approach facilitated a rigorous and systematic interpretation of the data, reducing bias and providing a nuanced understanding of healthcare professionals’ experiences and perceptions regarding obesity management training [22,27,28]. Themes and subthemes are summarized in Fig. 4.

## 3 Results

### 3.1 Survey

100 healthcare professionals responded the survey, their demographics characteristics are summarized in Table 1, that illustrates the range of professionals identified by participants as members of their multidisciplinary obesity management teams. Of the total respondents, 88 reported currently treating patients with obesity in their clinical practice. This subgroup proceeded to complete the remainder of the survey.

**Table 1:** Participants demographic – Survey.

Profession	Dietitian	Doctor	Others					
			Nurse	Health Coach	Physiotherapist	Nutritionist	Psychologist	Total
	55	20	13	5	3	3	1	100
Gender								
Male	1	14	0	2	1	0	1	19
Female	54	6	13	3	2	3	0	81
Age								
<35	15	1	7	2	0	2	0	27
35-49	25	14	3	2	1	1	0	46
>50	15	5	3	1	2	0	1	27
Do you treat people living with obesity?								
Yes	45	20	13	5	2	2	1	**88**
No	10	0	0	0	1	1	0	12
How many years of experience do you have working in weight management?								
>10 years	23	7	2	0	0	0	0	32
5–10 years	7	1	3	2	0	1	1	15
1–5 years	11	10	3	3	2	1	0	30
<1 year	4	1	4	0	0	0	0	9
No specified	10	1	1	0	1	1	0	14
Area of work								
Private	10	6	4	3	0	1	1	25
Public	31	10	7	1	1	1	0	51
Skipped	14	4	2	1	2	1	0	24
Do you work as part of a multidisciplinary team ?								
Yes	36	15	10	3	0	2	1	67
No	5	1	1	1	1	0	0	9
Skipped	14	4	2	1	2	1	0	24

Out of the 67 participants who reported working as part of a multidisciplinary team (MDT), 20 % indicated working with a dietitian and 20 % with a nurse. Additionally, 15 % reported collaborating with a physician, 12 % with a surgeon, 11 % with a psychologist, and 8 % with a physiotherapist. Smaller proportions of participants reported working with a health coach (4 %) and a sport physiotherapist (2 %). Another 8 % mentioned working with other professionals, including an occupational therapist, social worker, consultant psychiatrist, healthcare assistant, pediatric consultant, pharmacist, and psychology assistant.

#### 3.1.1 Training

Fig. 1A, Fig. 1BA and B reflect responses to questions regarding whether participants had received training in weight management during their undergraduate program and/or through postgraduate training, how they rated the effectiveness of this training.

**Fig. 1A:**
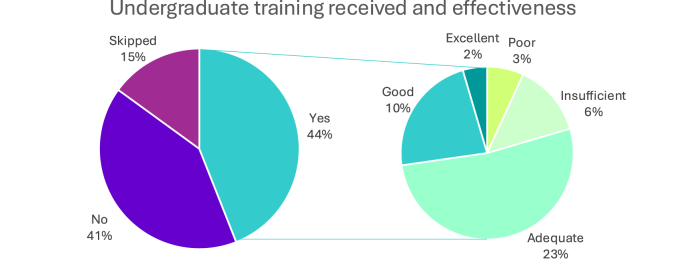
Weight management training in degree program and its effectiveness.

**Fig. 1B:**
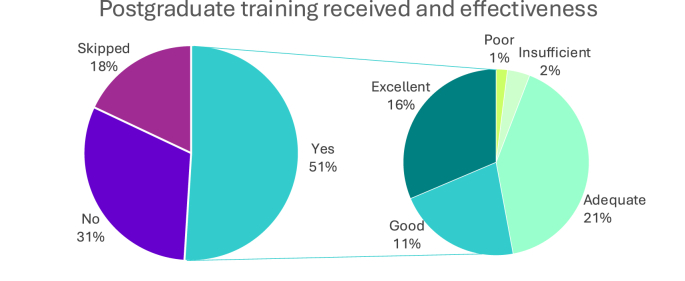
Postgraduate weight management received and its effectiveness.

When participants were asked about the type of training they received and the specific areas covered, they were allowed to select multiple responses, which shows that many professionals had undergone more than one form of training.

It was found that 5 % of participants reported receiving formal training through a master's degree and 1 % through a higher diploma, while 21 % received online training. Other participants received informal training 21 % through attending conferences, lectures, or workshops, and 19 % through independent study or reading. On-the-job training by a weight management professional was reported by 13 %, while 19 % received training through “other methods”, including PhD program, continued professional development (CPD), and obesity research fellowships.

In terms of areas of training 29 % received training in nutrition and another 29 % in awareness of obesity bias and stigma. Pharmacotherapy management was covered by 20 % of participants, bariatric surgery care by 18 %, physical activity by 13 %, and 2 % reported training in “other areas”, including psychological aspects, trauma, and mental health.

#### 3.1.2 Confidence and perceived gaps

When participants were asked how often they felt their current knowledge of obesity management was insufficient, 3 % reported "always," 22 % said "usually," 37 % "sometimes," 22 % "rarely," and 2 % "never," while 14 % skipped the question.

Regarding challenges in managing obesity due to lack of training or resources, 8 % of participants reported "always," experiencing them, 9 % "usually," 47 % "sometimes," 20 % "rarely," and 2 % "never," with 14 % skipping the question.

In terms of the perceived impact of further obesity management training on clinical care delivery, 20.3 % of participants believed it would be "extremely" helpful, 31.8 % "significantly," 22.7 % "moderately," and 10.2 % "slightly," while 1.1 % felt it would not help at all and 13.6 % skipped the question.

Fig. 2A presents the results for the areas in which participants felt most confident, while Fig. 2B shows the areas where participants reported having no confidence. Participants were allowed to select multiple responses, reflecting the variability in confidence across different domains of practice. Fig. 3 illustrates participants’ confidence levels across various aspects of obesity care, including overall management, diagnosis, and different treatment options.

**Fig. 2A:**
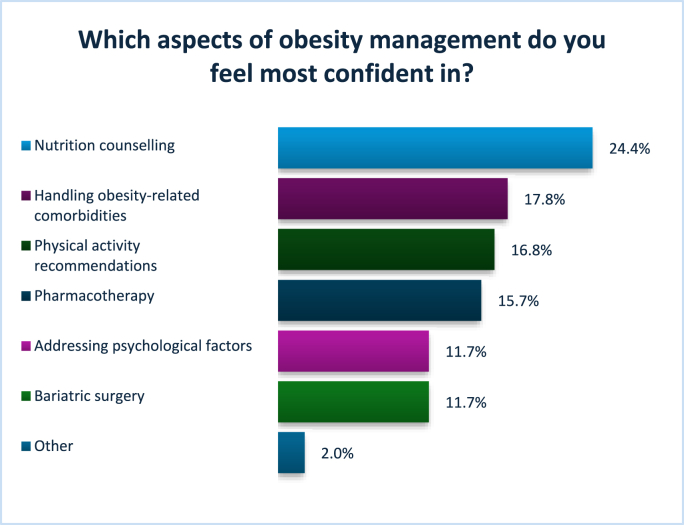
Areas of most confidence Other: disordered eating, individualized care, and healthy lifestyle advice.

**Fig. 2B:**
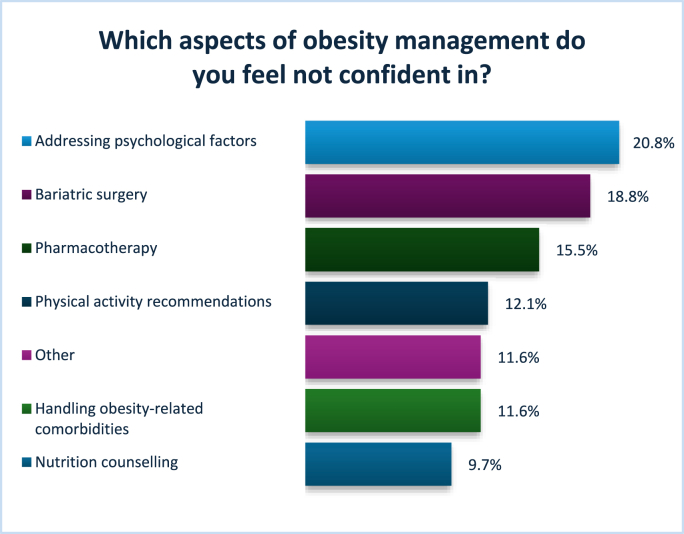
Areas of no confidence Other: trauma, BMI>70kg/m2, EOSS stage 4, eating disorders, complex binge eating.

**Fig. 3:**
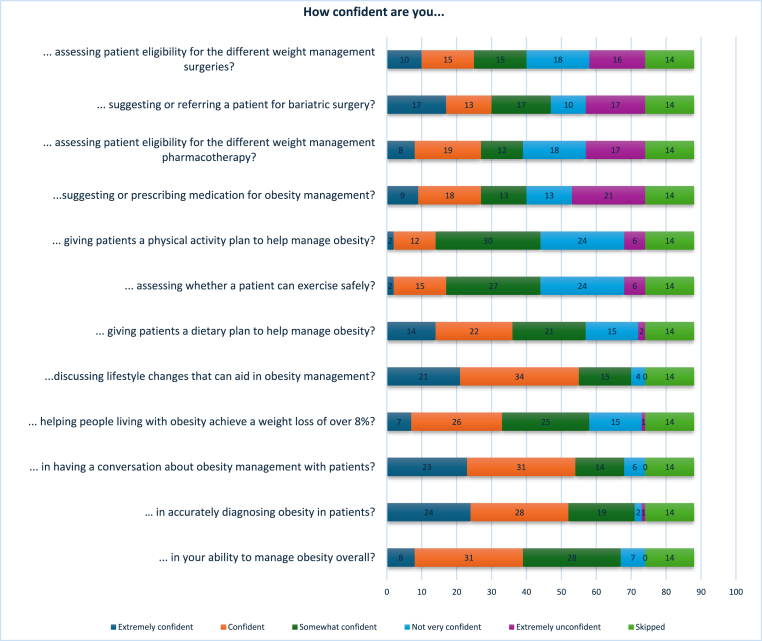
Confidence levels reported by participants across multiple tasks related to obesity management.

**Fig. 4:**
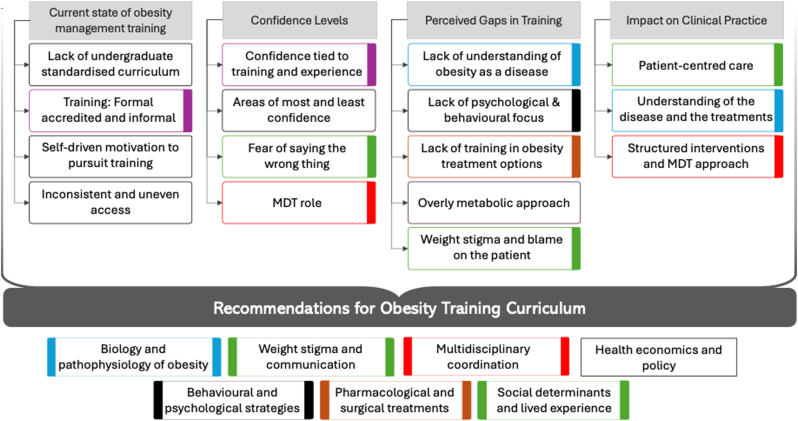
Identified domains reflecting perceptions of obesity training. Each domain comprises related themes, visually grouped by consistent color coding. These themes collectively inform curriculum recommendations for obesity management training. Identified gaps align directly with these recommendations, emphasizing key areas for improvement.  – State and confidence in training.  – Weight stigma and communication.  – Multidisciplinary (MDT) coordination.  – Biology and pathophysiology of obesity. **Black** – Behavioral and psychological strategies.  – Obesity treatment. Themes not color-coded were mentioned but not thematically linked. *Health economics and policy* was cited as an important recommendation, not tied to a specific domain. (For interpretation of the references to color in this figure legend, the reader is referred to the Web version of this article.)

When the same questions were separated and analyzed by profession, clear differences in confidence levels across various areas of obesity management emerged. Dietitians reported higher levels of confidence in providing dietary advice and discussing lifestyle changes to support weight management. They also felt relatively confident in helping patients achieve weight loss and in managing obesity overall. However, dietitians expressed the least confidence in areas related to pharmacological and surgical management, such as prescribing medication, assessing eligibility for weight management surgery, or referring patients for bariatric procedures.

In contrast, doctors reported the highest confidence in diagnosing obesity and managing obesity-related complications, as well as in using pharmacological treatments. They also showed moderate confidence in overall obesity management. However, doctors expressed lower confidence in areas involving behavioral and lifestyle interventions, particularly in delivering dietary guidance, creating physical activity plans, and supporting long-term lifestyle changes—areas where dietitians felt significantly more competent.

### 3.2 Semi-structured interviews

Twenty healthcare professionals participated in the interview, including 9 dietitians, 7 nurses, 3 doctors and 1 health coach.

Participant perceptions of obesity training were organized in 5 domains a) Current state of obesity management training, b) Confidence level, c) Gaps in training, d) Impact on clinical practice and d) Recommendation for obesity training curriculum. Each domain encompasses between 3 and 5 different themes which are reflection of the explicit meaning of the data, and 7 recommendations for further training. Fig. 4 the domains and themes are presented.

A) Current state of obesity management training

The first domain explored the state of obesity management training, based on participant's experience, where the main themes were:

Lack of undergraduate standardized curriculum: Participants found that obesity management is minimally covered during their early education and was not formally taught and only mentioned as a risk factor.

*‘We never really learned about how to treat obesity, just that it's a risk for diabetes’*

*'It was just mentioned alongside other conditions, not in-depth.'*

They described that there is a lack of obesity-specific modules, with few or no formal modules on obesity in their undergraduate's curricula.

*'There were no obesity modules at all in my undergraduate training.'*

*'Not one course really addressed how to help someone lose weight.'*

*'I didn’t see any structured learning on this until postgraduate level.'*

#### 3.2.1 Training

- Formal accredited training: Some of the HCP sought further formal training in obesity management, with access to structured training. SCOPE accreditation and Masters degree in Obesity were mentioned among the most common trainings received by the participants with positive experiences.

*'I did the SCOPE training, it was helpful and well-structured, I had modules on communication which really helped with patient engagement.'*

*'After doing the Master, I changed how I approached patients entirely. It gave me the tools I didn’t even know I was missing. I use what I learned from the Master’s in every consultation now.'*

*'The academic foundation from the Master gave me more authority when making clinical decisions. It was expensive but worth every penny because it directly improved my practice.'*

- Informal training: Participants also mention alternative forms of education as a source and foundation of their training, either one-off events such as webinars and conferences or on-the-job training such as mentorship-based learning and shadowing other senior colleagues.

*'These online talks are where I’m getting most of my education now, I went to a couple of webinars on weight stigma and management.'*

*'Most of what I know came from watching senior colleagues, my consultant guided me a lot in obesity management.'*

Self-driven motivation to pursuit training: Participants independently and voluntarily sought training by their own motivations, self-funding them, and driven by personal interest. They reflect a lack of support by their institutions.

*‘I did the master's in obesity because I knew I needed it, no one asked me to—I just knew it would help my patients.'*

*'I had to look for this training myself, I paid for it out of pocket because it wasn’t offered.'*

Inconsistent and uneven access: Training opportunities vary significantly depending on institutions. Access is inconsistent across locations and organizational priorities.

*'Depends where you work—some places care more than others. My colleague in a different trust got full training—I didn’t, it’s unfair.'*

*'It’s very ad-hoc, there are no system to make sure we all get trained, webinars and CPD events are all we really get offered, they are useful, but not the same as real training … it’s very much piecemeal—it depends what’s available that month.'*

B) Confidence levels

The second domain exposes the confidence level of participants and the impact of education in obesity on it. Four themes were found:

Confidence tied to experience and training: Clinicians who received formal training felt more equipped, confident, and recognized the importance of educational programs to increase confidence. Also, they recognized that low confidence levels were more prominent in those who lacked training. Moreover, hands-on exposure, repetition, and case-based learning boosted confidence and built competence.

*'Education builds confidence—it’s that simple.’*

*‘I’m much more confident now that I’ve done training, without training, I’d be guessing what to do.'*

*‘With experience, you start to know what works and what doesn’t, being in the clinic every day really helped me pick up skills.'*

Areas of most and least confidence: Participants have higher confidence in areas such as physical health markers, biomedical structured areas, and giving general advice. However, confidence was lacking when discussing sensitive topics and emotion-driven eating. In other words, their communication skills and long-term weight planning.

*'I’m confident explaining BMI, lab results, I know how to explain the clinical side of obesity.'*

*'I never know the right words when the topic gets sensitive.'*

*'How do you deal with relapse? That’s not something we’re taught.'*

Fear of saying the wrong thing: Participants highlighted the challenge of using the appropriate language when talking about obesity with patients. This fear of offending patients leads to avoidance of weight discussions.

*'It’s a hard topic to bring up, especially without tools, I avoid it if I don’t know how to say it properly.'*

*'I don’t want to damage trust by saying the wrong thing.'*

MDT role: Professionals are unclear on boundaries of their role vs. other HCPs of the team. There are unclear responsibilities, and they hesitate when starting a referral.

*'I’m not sure when I should refer or handle it myself? Do I talk about it, or is it the dietitian’s role? We need better role definitions'*

*'We overlap with other specialties and it’s confusing.'*

C) Perceived gaps in training

The third domain explores the HCP's perceptions about the gaps in the training received, in which five main themes were identified:

Lack of understanding of obesity as a disease: Participants found that obesity is not covered as a chronic, relapsing disease, and its pathophysiology and biological complexity is often ignored and not taught.

*'We don’t get taught that obesity is a disease, more like a side effect, I didn’t learn anything about the biology behind it.'*

*'It’s presented as a lifestyle issue, not a medical one, we need to understand obesity like we do diabetes or asthma.'*

Lack of psychological & behavioral focus: Participants received minimal training in behavior change and felt underprepared to address emotional and behavioral components.

*'There’s a lot going on emotionally and we don’t cover that.'*

*'We don’t learn motivational interviewing unless we seek it out.'*

Lack of training in obesity treatment options: Clinicians felt unaware of the most innovative pharmacological and surgical options; their training has lacked exposure to most modern evidence-based treatments.

*'I had no idea surgery was even considered unless it was extreme.'*

*'There was nothing about pharmacological support for obesity, we never covered the newer medications like GLP-1s.'*

Overly metabolic approach: HCP found that the training has a high medical/metabolic focus in isolation of the social and psychological context of the patient.

*'Training was all about insulin resistance and fat metabolism.'*

*'We learn about obesity like it’s a math problem, social context? Hardly touched.'*

Weight stigma and blame on the patient: Participants acknowledged that weight stigma remains prevalent within professional settings and expressed concern that current training does not adequately prepare them to address stigma or avoid stigmatizing language and bias. They highlighted that undergraduate education often reinforces the "willpower narrative", suggesting that weight loss is solely a matter of lifestyle choices and personal responsibility, thereby neglecting the complex, multifactorial nature of obesity.

*'Stigma exists even among clinicians and we’re not addressing it.'*

*'Obesity was presented as poor lifestyle choices, nothing more, it was always just ‘eat less and move more’—like it was that simple’*

D) Impact on clinical practice

The fourth domain explores the impact of obesity training on clinical practice, highlighting how clinicians’ approaches have been shaped by their educational experiences. Four key themes were identified within this domain:

Patient-centered care: Participants recognized how further education has improved their ability to talk about weight empathetically and without blame on the patient.

*'I use much more empathetic language now.'*

*'Patients are more responsive when you speak without judgment, at the end of the day it’s not about blame, it’s about support.'*

Clinicians after training have learned how to focus on what matters to the patients, improving their engagement in the treatment since goals are aligned with their values and expectation.

*'I ask what matters to them before suggesting goals, it’s important they feel ownership over their goals.'*

*'I learned how to focus more on their personal motivation.'*

Understanding obesity as a disease and the treatments available: HCP reported that gaining a deeper understanding of obesity as a chronic, relapsing disease led to a significant shift in their clinical approach. This knowledge encouraged a move away from judgmental attitudes towards more empathetic, supportive care, motivating them to seek appropriate, evidence-based treatment options for their patients.

*'Once I understood obesity as a disease, it changed everything, I stopped blaming the patient and focused on the biology and support, and now I explain it like I would with diabetes or hypertension.'*

Structured interventions and MDT approach: Trained professionals reported using structured, individualized, and goal-oriented care plans, moving away from vague or generalized advice. Training facilitated more effective collaboration with other team members, enhancing integration within multidisciplinary teams and improving clinicians’ sense of teamwork and coordinated care delivery.

‘*Now I give people actual steps, not just 'lose weight’, I work with them to create goals together—it’s more realistic, it’s structured, not vague like before.'*

*'Multidisciplinary care is more effective so I don’t feel like I have to do it all alone anymore.'*

*'Training showed me how to use the whole team, I bring in dietitians and psychs more now.'*

E) Recommendations for obesity training curriculum

The fifth and final domain focused on participants' recommendations regarding essential content for a Master's program in obesity management. Interviewees were asked to imagine themselves as program directors and identify the top priority topics they would include to better prepare healthcare professionals for clinical practice in obesity care. Their responses highlighted key areas they believed were essential for comprehensive training. Seven themes emerged from their suggestions:

Biology and pathophysiology of obesity: Participants emphasized the need for deeper scientific understanding of obesity. They highlighted the need for comprehensive coverage of metabolic, hormonal, and neurobiological mechanisms contributing to the development and progression of the disease, recognizing this knowledge as foundational for evidence-based obesity management.

*'Understanding the science helps break the idea that it’s just willpower, we need a proper module just on the biology of obesity, which would prepare us better to explain to patients how their hormones and brain are involved.'*

Behavioral and psychological strategies: Professionals requested more structured content on how to support long-term behavior change and use psychological tools such as motivational interviewing.

*'Knowing how to help someone long-term is a skill we’re not given, we need more about motivational interviewing and behavior change.'*

Weight stigma and communication: Clinicians emphasized the need for training in using sensitive, non-stigmatizing language, addressing bias and improving patient communication.

*'Stigma should be a core topic—it’s everywhere.'*

*'We don’t get trained on how to talk without blame, bias and assumptions are never really addressed in our learning.'*

Pharmacological and surgical treatments: Lack of training in modern treatment options led to recommend modules on pharmacotherapy and bariatric surgery to enhance understanding and application of evidence-based interventions.

*'No one teaches you about the treatment algorithm for obesity, even the basics of pharmacotherapy were missing in my training.'*

*'I want modules on meds—what to use, when, and how. GLP-1s and other options should be standard content.'*

*'We need clarity on when surgery is appropriate and how to refer.'*

Multidisciplinary coordination: There was a call for better understanding of each profession's role in obesity management and how to collaborate effectively, clarifying roles and enhancing teamwork.

*'It would help to actually train with other disciplines, I want to understand what a dietitian does so I can complement it.'*

Social determinants and lived experience: Participants advocated for greater emphasis on the real-life challenges faced by people living with obesity. They recommended incorporating patients' lived experiences and social contexts into training to foster empathy, reduce stigma, and promote more holistic, person-centered care.

*'Patients need to be part of the curriculum—we should hear from them.'*

*'We talk about food and exercise, but not poverty or trauma, it’s not just biology; it’s life circumstances too, we need to know more on health inequality and environment, it would make a real difference.'*

Health economics and public policies: Participants highlighted the need to understand cost-effectiveness, policy frameworks, and the health system impact of obesity care to better advocate for resources and contribute to effective service design.

*'We should learn about what interventions save money long-term.'*

*'Understanding the economics of obesity would help us influence change, justify the services we provide, we don’t understand the cost side—what’s worth funding and why.'*

## 4 Discussion

The findings from this study provide essential insight into the current status of obesity management training among healthcare professionals working in obesity management in active clinical practice. Of the 100 clinicians surveyed, 88 reported working directly in obesity care, with the majority having more than five years of experience. Most (n = 67) practiced within MDTs, while others, predominantly dietitians, worked independently, highlighting variations in access to collaborative support structures.

Participants reported receiving training in obesity management during undergraduate or postgraduate education, yet the nature of this training was largely informal, comprising experiential learning, personal study, and participation in continuing professional development activities such as webinars and conferences. Only a small proportion had completed structured, formal education on obesity. This reflects a broader international pattern. In the United States, a national study by Butsch et al. revealed that medical schools allocated minimal curriculum time to obesity, with only 27 % of institutions offering any dedicated training, leading to widespread deficiencies in student preparedness [7]. Similarly, a Canadian survey by Katz et al. found that both students and medical school deans recognized significant inadequacies in obesity training, particularly regarding behavioral interventions and pharmacotherapy [29]. A global systematic review by Mastrocola et al., covering medical education in 27 countries, found that obesity content was poorly integrated, rarely competency-based, and often dependent on individual educator interest rather than institutional policy [8]. In the UK, Chisholm et al. observed that medical schools frequently failed to meet the General Medical Council's guidelines on lifestyle and obesity education, noticing inconsistent teaching and lack of trained faculty as key barriers [30].

Although most respondents in this study rated their training as adequate, good, or excellent, substantial gaps emerged. These included limited knowledge of obesity's pathophysiology, lack of behavioral and psychological intervention training, and insufficient familiarity with pharmacological and surgical options. Similar deficits have been identified by other authors. Jay et al. reported that physicians with limited training were less confident in their ability to provide effective obesity care and more likely to hold stigmatizing beliefs [10]. In a review by Kushner and Ryan, knowledge gaps in core lifestyle management strategies, including behavioral counselling and treatment planning, were shown to undermine clinical effectiveness [11]. In Canada, Stanford et al. found that physicians who had not received formal obesity training in medical school or residency were significantly less knowledgeable about bariatric surgery, aligning with our findings of low confidence in this domain [13].

When analyzing confidence levels across different areas of obesity management, participants overall reported the highest confidence in nutritional counselling and managing obesity-related comorbidities. This pattern appears to be strongly influenced by the professional composition of the sample, which included a higher proportion of dietitians, doctors, and nurses—professions typically more engaged in these aspects of care. However, when the same questions were stratified by profession, distinct differences in confidence emerged.

Dietitians reported the greatest confidence in delivering dietary advice and discussing lifestyle modifications, which aligns closely with their core scope of practice. They also felt relatively confident in supporting patients to achieve weight loss and in managing obesity overall. Conversely, dietitians expressed notably lower confidence in pharmacological and surgical aspects of obesity care, such as assessing eligibility for anti-obesity medications or bariatric surgery and making related referrals. Doctors, on the other hand, were most confident in diagnosing obesity and managing related complications, as well as in the use of pharmacotherapy. While they reported moderate confidence in overall obesity management, they consistently expressed lower confidence in providing dietary counselling, designing physical activity plans, and supporting behavioral change—areas where dietitians felt more competent. These results align with findings from Antognoli et al., who noted that training in obesity-related communication, nutrition, and physical activity was inconsistent across primary care residencies in the United States [14]. Similarly, Chisholm et al. demonstrated that medical students in the UK were more confident performing biomedical assessments than delivering lifestyle advice, due to a lack of skills-based training [31]. These findings highlight profession-specific strengths and gaps, reinforcing the value of interdisciplinary collaboration.

Interestingly, discrepancies emerged between survey and interview data regarding confidence levels. While participants expressed low confidence in areas such as pharmacotherapy, exercise safety, and bariatric referral pathways in the survey, interview narratives suggested higher comfort with biomedical tasks, such as interpreting clinical markers and giving general advice. This mismatch may reflect role ambiguity within MDTs. Interviewees frequently cited uncertainty about their professional remit and how best to collaborate with colleagues, issues also raised by the Health Service Executive's (HSE) national Model of Care for obesity, which emphasizes the need for clear role definitions and structured team integration to improve patient outcomes [32]. Koch et al. have also underscored the importance of formally organized training pathways to ensure consistent interprofessional competence in bariatric and obesity care [33].

The clinical implications of these educational gaps are significant. In this study, 61 % of respondents indicated that they sometimes, usually, or always felt their knowledge in obesity management was insufficient. 64 % reported challenges in delivering care due to inadequate training or limited resources, while 75 % believed that further education would improve their ability to provide high-quality patient-centered care. These concerns mirror those of US internal medicine residents surveyed by Butsch et al., who overwhelmingly felt unprepared to manage obesity, citing lack of curriculum time and formal instruction as primary factors [34]. Similarly, a study by Simon and Lahiri found that clinicians in large healthcare systems reported low confidence and high perceived barriers to delivering obesity care, including knowledge deficits and uncertainty about treatment options [35].

The curriculum recommendations made by participants in this study reflect both the perceived deficits and a clear demand for more structured and comprehensive training. The areas suggested—obesity pathophysiology, behavioral and psychological interventions, communication and weight stigma, pharmacological and surgical treatment options, MDT coordination, social determinants and lived experience, and health policy—closely align with established international educational frameworks. For instance, Obesity Canada's competency framework includes core elements such as addressing weight bias, integrating lived experience, and applying a biopsychosocial approach to care [20]. Similarly, the Obesity Medicine Education Collaborative (OMEC) in the United States incorporates competencies in clinical assessment, patient-centered communication, and evidence-based treatment planning [19]. These frameworks are further supported by the World Health Organization's Acceleration Plan to Stop Obesity, which emphasizes the need to train healthcare professionals to address social inequities, stigma, and environmental contributors to obesity [21].

Ultimately, these findings reinforce the need for a systematic, multidisciplinary, and standardized approach to obesity training across all stages of health professional education, and most importantly for professionals working on obesity management services. Embedding these components into formal curricula can address knowledge deficits, reduce stigma, promote greater clinical confidence, and improve patient's outcomes.

## 5 Strengths and limitations

A key strength of this study lies in its convergent mixed-methods design, which enabled a comprehensive exploration of obesity education by combining the breadth of survey data with the depth of qualitative interviews. This approach is particularly well-suited to complex educational topics, offering both generalizable trends and in-depth contextual insights. The sample size, 100 survey respondents and 20 interviewees, is considerable relative to the size of the obesity management workforce in Ireland and includes a wide range of healthcare professionals, such as dietitians, doctors, nurses, psychologists, physiotherapists, and health coaches, reflecting multidisciplinary perspectives.

The qualitative analysis was further strengthened by the fact that two researchers independently conducted the thematic coding, reducing potential bias and enhancing the rigor of interpretation. In addition, interviews were conducted until data saturation was reached, ensuring comprehensive coverage of recurring themes and issues.

However, some limitations must be acknowledged. Participant recruitment was facilitated through the authors' professional networks and the Association for the Study of Obesity on the Island of Ireland (ASOI), which may have led to an overrepresentation of dietitians. This likely reflects both ASOI's active engagement in obesity-related work and the longstanding involvement of dietitians in obesity management. Additionally, the voluntary nature of participation may have introduced selection bias, as individuals with particularly strong views or more direct experience in obesity care may have been more inclined to take part. This could limit the representativeness of the findings.

Certain healthcare professional groups may have been underrepresented, potentially affecting the breadth and generalizability of the results across the wider healthcare workforce. Furthermore, all data were self-reported, which may be subject to recall bias or social desirability bias. Lastly, the quantitative data were analyzed descriptively only, which limits the depth of statistical inference that can be drawn from the findings.

## 6 Conclusion

This study identifies critical gaps in training among healthcare professionals working in obesity management in a European country such as Ireland, with many participants reporting insufficient preparation in key areas such as behavioral interventions and pharmacotherapy. Confidence levels varied by profession and reflected the limitations of current training pathways. Weight stigma and role ambiguity within multidisciplinary teams further impacted the quality of care.

Participants recommended curriculum areas such as pathophysiology, behavioral strategies, communication, and stigma reduction, which align with recommendations from the World Health Organization, who call for integrated, competency-based obesity education. Embedding structured, evidence-based training is essential to improve confidence, reduce bias, and effectively support obesity care across multidisciplinary healthcare settings.

## Key takeaway massages

• Obesity education is inconsistent and often informal, leaving professionals underprepared in key areas like behavior change and pharmacotherapy.
• Confidence varies by profession, highlighting the need for clearer roles and stronger interdisciplinary collaboration.
• There's strong demand for formal, competency-based training to improve confidence, reduce stigma, and support better care.

## Ethical considerations

Ethical approval was obtained from the Research Ethics Committee (REC), reference number: LS-24-69-Najim, University College Dublin, Ireland. Informed consent was obtained from all individuals participants included in the study.

## Authors contribution

FC: writing-review & editing, conceptualization, methodology, data analysis, visualization. L.K: data analysis, writing-review and editing WAN: supervision, conceptualization, methodology, writing-review and editing, CWlR: supervision, writing-review and editing. All authors approved the final version.

## Data availability

The data generated during and analyzed during the study are available from the corresponding author on reasonable request.

## Declaration of Artificial Intelligence (AI) and AI-assisted technologies

Artificial intelligence tools were used solely to assist with grammar correction during the preparation of this manuscript. No AI tools were used for data analysis, interpretation, or content generation. The authors reviewed and edited the content as needed and take full responsibility for the content of the publication.

## Funding

This project is funded by the European Commission's Erasmus Mundus Design Measure grant number 101128158.

## Declaration of competing interest

ClR reports grants from the Irish Research Council, Science Foundation Ireland, Anabio, and the Health Research Board. He serves on advisory boards of Novo Nordisk, Herbalife, GI Dynamics, Eli Lilly, Johnson & Johnson, Glia, Keyron, and Boehringer Ingelheim. ClR is a member of the Irish Society for Nutrition and Metabolism outside the area of work commented on here. He was the chief medical officer and director of the Medical Device Division of Keyron in 2021. Both of these are unremunerated positions. No patients have been included in any of Keyron's studies and they are not listed on the stock market. ClR was gifted stock holdings in September 2021 and divested all stock holdings in Keyron in September 2021. He continues to provide scientific advice to Keyron for no remuneration. Other authors have no conflicting interests to declare that may influence their judgments on what is published.

## Appendix A Supplementary data

Supplement 1 – Questionnaire.

Supplement 2 - Semi structured interviews.
