# Supplementary material for: Healthcare professionals’ perspectives on the current state of obesity management training: A mix methods study
Source: Obes Pillars. 2025 Aug 19;16:100202. doi: 10.1016/j.obpill.2025.100202 (PMC12408399; doi:10.1016/j.obpill.2025.100202)
Supplement: Multimedia component 1 [file mmc1.docx]

| **Appendix 1 – Questionnaire** | |
| --- | --- |
| **Demographic**   1. Gender:  - Male - Female - Other: __________  1. Age  - < 35 years old - 35 – 49 years old - >50 years old  1. Profession  - Doctor - Nurse - Dietitian - Physiotherapist - Psychologist - Other  1. Do you treat people with obesity in your area of practice ?  - Yes - No  1. How many years of experience do you have working in weight management ?  - < 1 year - 1 – 5 years - 5 – 10 years - > 10 years | **Current obesity management training**   1. Did you receive training in weight management during your degree program ?  - Yes - No   1. If you received training, how would you rate its effectiveness? - Excellent: Thorough and highly comprehensive - Good: Provided complete and useful information - Adequate: Covered most of the necessary topics - Insufficient: Lacked some important details - Poor: Incomplete and unhelpful  1. Have you received specific training in weight management after finishing your degree program?  - Yes - No   1. If yes, What type of training did you received in weight management - Master degree __________________ - Higher diploma - Online training __________________ - Conference, Lecture, Workshop, Seminar - On the job training by a weight management professional - Independent study/reading - Other ________________________   1. If yes, how would you rate its effectiveness? - Excellent: Thorough and highly comprehensive - Good: Provided complete and useful information - Adequate: Covered most of the necessary topics - Insufficient: Lacked some important details - Poor: Incomplete and unhelpful   1. If yes, in which specific areas have you received training (select all that apply) - Nutrition - Physical activity - Pharmacotherapy management - Bariatric surgery care - Awareness of obesity bias and stigma - Other:__________________  1. Do you follow any obesity management national or international guidelines?  - Yes (specify) ________________ - No |
| **Obesity settings**   1. Do you work in  - Public sector - Private sector  1. Do you work as part of a multidisciplinary team ?  - Yes - No   1. If yes, which professionals are part of the multidisciplinary team ? (select all that apply ) - Doctor___________________ - Nurse - Dietitian - Physiotherapist - Psychologist - Health coach - Other ________________ |  |
| **Impact on clinical practice**   1. How often do you encounter challenges in managing obesity due to lack of training or resources  - Never - Rarely - Sometimes - Often - Always  1. How much do you believe that additional training in obesity management would improve your ability to deliver better patient care?  - Not at all - Slightly - Moderately - Significantly - Extremely | **Perceived gaps in knowledge and skills**   1. Which aspects of obesity management do you feel most confident in?  - Nutrition counselling - Physical activity recommendations - Pharmacotherapy - Bariatric surgery - Addressing psychological factors - Handling obesity-related comorbidities - Other: ________________  1. Which aspects of obesity management do you feel least confident in?  - Nutrition counselling - Physical activity recommendations - Pharmacotherapy - Bariatric surgery - Addressing psychological factors - Handling obesity-related comorbidities - Other: ________________  1. How often do you feel your current knowledge of obesity management is insufficient to address the needs of your patients?  - Never - Rarely - Sometimes - Often - Always |
| **Confidence**   \| How confident are you \| Extremely confident \| Confident \| Somewhat confident \| Not very confident \| Extremely unconfident \| \| --- \| --- \| --- \| --- \| --- \| --- \| \| **Managing obesity** How confident are you in your ability to manage obesity overall? \|  \|  \|  \|  \|  \| \| **Diagnosing obesity** How confident are you in accurately diagnosing obesity in patients? \|  \|  \|  \|  \|  \| \| **Discussing obesity management** How confident are you in having a conversation about obesity management with patients? \|  \|  \|  \|  \|  \| \| **Achieving clinically significant weight loss (>8%)** How confident are you in helping people living with obesity achieve a weight loss of over 8%? \|  \|  \|  \|  \|  \| \| **Discussing lifestyle modifications** How confident are you in discussing lifestyle changes that can aid in obesity management? \|  \|  \|  \|  \|  \| \| **Providing a dietary plan** How confident are you in giving patients a dietary plan to help manage obesity? \|  \|  \|  \|  \|  \| \| **Assessing ability to exercise safely** How confident are you in assessing whether a patient can exercise safely? \|  \|  \|  \|  \|  \| \| **Providing an activity plan** How confident are you in giving patients a physical activity plan to help manage obesity? \|  \|  \|  \|  \|  \| \| **Recommending pharmacotherapy for obesity management** How confident are you in suggesting or prescribing medication for obesity management? \|  \|  \|  \|  \|  \| \| How confident are you assessing patient eligibility for the different weight management pharmacotherapy ? \|  \|  \|  \|  \|  \| \| **Recommending bariatric surgery** How confident are you in suggesting or referring a patient for bariatric surgery? \|  \|  \|  \|  \|  \| \| How confident are you assessing patient eligibility for the different weight management surgeries ? \|  \|  \|  \|  \|  \| | |
| Would you be open to being contacted for a follow-up interview with the researcher to explore your knowledge and experience in greater detail?   - Yes - No | |

| **Appendix 2 – Semi structured interviews**  **Section 1: Introduction and Current Training in Obesity Management**   1. Can you briefly describe your role and responsibilities in obesity management within your practice? 2. What type of training did you receive before starting work in the weight management service? 3. What type of training have you received during your time working in the weight management service?    - Have you pursued any additional or specialized training since then?    - If yes, What motivated you to seek additional training in obesity management? 4. Reflecting on the obesity management training you’ve received, how well do you feel it prepared you for the challenges you face in clinical practice?    - What elements of the training were most valuable? 5. What gaps or limitations did you encounter in the training?    - Were there any particular topics that you felt were missing or underemphasized? 6. Have you had any experiences where the training you received significantly impacted patient outcomes? 7. Do you feel that the training materials and resources are up-to-date with current best practices in obesity management?    - If not, in what areas do you think they are outdated?   **Section 2: Perceived Gaps in Knowledge and Skills**   1. Which areas of obesity management do you currently feel least confident in? Why? 2. Have you encountered situations where you felt your knowledge or skills were insufficient to provide optimal care?    - What resources or support would have helped in these situations?   **Section 3: Impact on Clinical Practice**   1. Can you describe a situation where a gap in your obesity management training affected your clinical decision-making?    - What could have been done differently with better training? 2. How do you think additional training in obesity management would improve your clinical practice?    - Which specific areas of training do you think would make the most impact? 3. What changes would you like to see in obesity management training to make it more effective for healthcare professionals?   **Section 4: Confidence in Obesity Management**   1. How confident do you feel in your ability to manage obesity overall? 2. Can you discuss any specific aspects of obesity management where you feel extremely confident? Is there a particular training or experience that has strengthened your skills in this area? 3. Conversely, are there aspects of obesity management where you feel less confident?    - What would help you improve in these areas?   **Section 5: Closing Thoughts**   1. If you had the opportunity to design an obesity management training program, what would be your top three priorities? 2. Is there anything else you’d like to add that we haven’t covered regarding obesity management training and practice? |
| --- |
